# Age-adjusted Charlson Comorbidity Index as a prognostic factor for radical prostatectomy outcomes of very high-risk prostate cancer patients

**DOI:** 10.1371/journal.pone.0199365

**Published:** 2018-06-20

**Authors:** Jae Won Park, Dong Hoon Koh, Won Sik Jang, Joo Yong Lee, Kang Su Cho, Won Sik Ham, Koon Ho Rha, Woo Hee Jung, Sung Joon Hong, Young Deuk Choi

**Affiliations:** 1 Department of Urology, National Health Insurance Service Ilsan Hospital, Goyang, Korea; 2 Department of Urology, Urological Science Institute, Yonsei University College of Medicine, Seoul, Korea; 3 Department of Pathology, Yonsei University College of Medicine, Seoul, Korea; University of South Alabama Mitchell Cancer Institute, UNITED STATES

## Abstract

**Purpose:**

Prostate cancer (PC) is a devastating and heterogeneous condition with diverse treatment options. When selecting treatments for patients with very high-risk PC, clinicians must consider patient comorbidities. We investigated the efficacy of the age-adjusted Charlson Comorbidity Index (ACCI) as a prognostic factor for patient outcomes after radical prostatectomy (RP).

**Materials and methods:**

We retrospectively investigated the medical records of PC patients at our institution who underwent RP from 1992 to 2010. Very high-risk PC was defined according to National Comprehensive Cancer Network guidelines. Patients with incomplete medical records or who had received neoadjuvant therapy were excluded. Preoperative comorbidity was evaluated by the ACCI, and the prognostic efficacy of the ACCI was analyzed using univariable and multivariable Cox regression, competing risk regression model and Kaplan-Meier curves.

**Results:**

Our final analysis included 228 men with a median age of 66 years (interquartile range 62–71) and median prostate specific antigen of 10.7 ng/mL. There were 41 (18%) patients with an ACCI score >3 and 88 (38.6%) patients with a biopsy Gleason score >8. Preoperative evaluation revealed that 159 patients (69.7%) had a non-organ confined tumor (≥T3). Following RP, 8-year prostate cancer-specific survival (PCSS) and overall survival (OS) rates were 91.6% and 83.4%, respectively. Competing risk regression analysis revealed that ACCI was significantly associated with other-cause survival and OS (p<0.05).

**Conclusion:**

The ACCI is an effective prognostic factor for other-cause survival and OS in very high-risk PC patients. RP should be considered carefully for patients with an ACCI score >3.

## Introduction

Prostate cancer (PC) is the most prominent newly-diagnosed cancer and the third highest cause of cancer-related deaths in the United States [1]. Because PC is highly prevalent and devastating, researchers have created a risk classification system based on clinical features of the disease. Previously, D’Amico et al. classified PC according to initial prostate-specific antigen (PSA) levels, clinical T stage, and biopsy Gleason score [2]. However, more recent studies indicate the need for a more stratified risk classification system [3]. National Comprehensive Cancer Network (NCCN) guidelines now separate high-risk PC into high- and very high-risk [4]. Given the heterogeneity of PC, diverse treatment options exist depending on disease severity. Radical prostatectomy (RP) is the one of the most widely accepted PC treatments. Although RP previously had adverse effects on urinary health, including urinary incontinence and sexual dysfunction [5], the introduction of robot-assisted surgical systems reduced the rate of RP-induced urinary complications [6]. More recent studies now describe RP as a feasible treatment for patients with locally advanced PC [7] and suggest that it may even be used to treat high-risk PC with similar therapeutic efficacy as other commonly used cancer treatments such as radiation and hormone combined therapy [8, 9].

When selecting from the diverse treatment options for PC, clinicians must consider the overall physical condition of patients, including their age and comorbidities. Comorbidity, in particular, is an important prognostic factor for patients with cancer [10]. The Charlson Comorbidity Index (CCI) is widely used to evaluate comorbidity in clinical practice [11]. In addition, age was also combined to CCI as an important prognostic factor, according to validation process [12]. In the context of PC, previous studies report an association between the CCI and survival in men with localized PC [13]. Current NCCN guidelines only recommend RP in young, healthy patients without tumor fixation to the pelvic sidewall [4]. However, RP may potentially be used to treat patients with very high-risk PC. In this study, we hypothesized that age adjusted CCI (ACCI) can predict outcomes following surgical treatment for patients with very high-risk PC. We retrospectively analyzed patient outcomes after RP for correlations between the ACCI and different measures of survival. Our results indicate that the ACCI may be used as a prognostic factor for survival after RP in patients with very high-risk PC.

## Materials and methods

Data were collected with approval from the Institutional Review Board at the Yonsei University College of Medicine (No. 4-2017-0864). We retrospectively reviewed the medical records of 4,440 patients who underwent RP at Yonsei University Health System from 1992 to 2010. For all patients analyzed, RP was performed by multiple surgeons using open or robot-assisted laparoscopic techniques. Patients with incomplete medical records or those who had received neoadjuvant therapy were excluded. Patients were classified into clinical risk groups according to NCCN guidelines. Very high-risk PC was defined as PC with clinical T3b or T4, a primary biopsy Gleason pattern of 5, a biopsy Gleason grade 5, and more than four biopsy cores with a Gleason grade of 4 or 5.[4] Tumor, node, and metastasis (TNM) stage was determined according to the American Joint Committee on Cancer 8^th^ edition.

Parameters recorded from these very high-risk PC patients included age, body mass index, initial PSA, prostate volume measured by transrectal ultrasonography, number of prostate biopsy cores, percentage of positive cores and pathologic features of the specimen at the time of RP. Additionally, biochemical recurrence (BCR)-free survival, PC-specific survival (PCSS), other-cause survival (OCS) and overall survival (OS) rates were analyzed. BCR was defined as subsequent detectable PSA values that increased at two or more time points.[4] Data regarding mortality and cause of death were obtained from the Yonsei Cancer Registry Center database at Severance Hospital. PC-specific mortality was defined as death after RP caused by PC or PC-related complications, whereas other-cause mortality was defined as deaths resulting from causes other than PC or complications of PC. Overall mortality was defined as death from any cause. Patient comorbidities were assessed by the ACCI, which was calculated from hospital discharge records.[11] The CCI included 19 conditions with scores assigned based on the severity of each disease. Beginning at the age of 50 years, one point was added to the CCI for every subsequent decade. PC was not included for the calculation of the CCI in this study.

Univariable and multivariable Cox regression analyses were performed on clinical parameters to investigate associations between different prognostic factors and OS. Competing risk regression model was also performed for PCSS and OCS with clinical parameters. Kaplan-Meier curves were used to compare PCSS, OCS and OS in two patient groups: high (>3) and low (≤3) ACCI groups. Octogenarian or septuagenarian prostate cancer patients are known to have poor overall survival after RP or radiotherapy, and ACCI scores for these patients total at least 3 by ACCI calculation formula.[14] Meanwhile, an ACCI score >3 has been shown to be associated with mortality risk in other malignancies.[15] In Surveillance, Epidemiology, and End Results (SEER) database, prostate cancer is most frequently diagnosed among men aged 65–74 years, and their ACCI score are 3 if they have no co-morbidity or minor co-morbidity.[16] Thus, we chose an ACCI score of 3 as a cut-off value.

All statistical analysis was performed using SPSS Statistics software, version 23.0 (IBM, Armonk, NY, USA) and R statistical software (version 3.1.0, R Foundation for Statistical Computing, Vienna, Austria; http://www.r-project.org), using the cmprsk and riskRegression packages for competing risk regression.

## Results

A total of 228 patients met the selection criteria and were included in this study. The median follow-up duration was 100 months. Descriptive statistics of patient demographic data and prostate characteristics are displayed in Table 1. The median age was 66 years (interquartile range [IQR] 62–71), and the median prostate volume was 30 mL (IQR 25–40). The median PSA level was 10.7 ng/mL (IQR 7.0–17.4). The median number of positive cores was five (IQR 2–7), and the median percentage of positive biopsy cores was 41.6% (IQR 20.0–66.7). Of these patients, 41 (18%) had an ACCI score >3. There were 88 (38.6%) patients with a Gleason score >8 at the time of prostate biopsy and 159 (69.7%) patients with clinical, non-organ confined PC (≥T3). After RP, these numbers increased to 120 (52.6%) patients with a Gleason score >8 and 179 (78.5%) patients with pathological, non-organ confined PC. Surgical margins were involved in 142 (62.3%) cases, with lymph node invasion diagnosed in 26 (11.4%) instances. Following RP, the 8-year BCR-free survival rate was relatively low at 44.1%. However, patient survival after RP was high, with 8-year PCSS and OS rates of 91.6% and 83.4%, respectively. There were 23 cases for PC-specific mortality and 23 cases for other-cause mortality during the follow-up period.

**Table 1:** Baseline characteristics.

	Median	IQR
Age, year	66	62–71
BMI, kg/m^2^	24.2	22.3–26.0
Prostate volume measured by TRUS, mL	30	25–40
PSA level, ng/mL	10.7	7.0–17.4
Number of positive core	5	2–7
Percentage of positive core, %	41.6	20.0–66.7
Tumor volume at specimen, cc	3	1.5–6.8
Biopsy Gleason score	N	%
≤8	140	61.4%
≥9	88	38.6%
Clinical T stage		
≤T2	69	30.3%
≥T3	159	69.7%
Pathologic Gleason score		
≤8	108	47.4%
≥9	120	52.6%
Pathologic T stage		
≤T2	49	21.5%
≥T3	179	78.5%
Positive surgical margin	142	62.3%
Pathologic LN metastasis	26	11.4%
Age adjusted Charlson Comorbidity Index		
≤3	187	82%
>3	41	18%
8Y BCR free survival rate		44.1%
8Y PC specific survival rate		91.6%
8Y Overall survival rate		83.4%

BCR = biochemical recurrence; BMI = body mass index; IQR = Interquartile range; LN = Lymph node; PC = prostate cancer; PSA = prostate specific antigen; TRUS = transrectal ultrasonography

Competing risk regression model was also performed for PCSS and OCS as displayed in Table 2. OCS was significantly associated with ACCI (HR 3.66, 95% CI 1.44–9.26). Competing risk regression analysis performed for PCSS revealed associations with a biopsy Gleason score ≥9 (HR 3.57, 95% CI 1.06–12.73), as reported in Table 3. In univariable competing risk regression analysis, only a single significant variable was observed. Therefore, we performed multivariable competing risk analysis using variables related to cancer characteristics. PSA level and clinical T stage were not associated with PCSS.

**Table 2:** Competing risk regression analysis for prostate cancer-specific survival and other-cause specific survival.

	Prostate cancer-specific survival				Other-cause survival			
	Univariable		Multivariable		Univariable		Multivariable	
	HR (95% CI)	p Value	HR (95% CI)	p Value	HR (95% CI)	p Value	HR (95% CI)	p Value
Age, year	1.06 (0.99–1.12)	0.06			1.10 (1.03–1.17)	<0.01	1.05 (0.98–1.12)	0.18
BMI, kg/m^2^	0.98 (0.78–1.23)	0.85			0.96 (0.79–1.17)	0.69		
PSA level, ng/mL	1.00 (0.98–1.01)	0.57	0.99 (0.98–1.01)	0.90	0.98 (0.96–1.01)	0.15		
Biopsy Gleason score		<0.01		0.04		0.69		
≤8	1 (ref)		1 (ref)		1 (ref)			
≥9	4.27 (1.64–10.75)		3.57(1.06–12.73)		0.84 (0.36–1.97)			
Number of positive biopsy core	0.95 (0.82–1.11)	0.55	1.02 (0.72–1.44)	0.92	0.98 (0.87–1.10)	0.67		
Percentage of positive biopsy core, %	0.99 (0.97–1.00)	0.15	0.99 (0.95–1.03)	0.48	0.99 (0.98–1.01)	0.46		
Clinical T stage		0.59		0.47		0.92		
≤T2	1 (ref)		1 (ref)		1(ref)			
≥T3	0.80 (0.34–1.82)		1.58 (0.45–5.51)		0.96 (0.39–2.32)			
ACCI		0.21				<0.01		<0.01
≤3	1(ref)				1(ref)		1(ref)	
>3	1.79 (0.72–4.47)				5.02 (0.23–11.30)		3.66 (1.44–9.26)	

**Table 3:** Univariable and multivariable Cox regression analysis for overall survival.

	Overall survival			
	Univariable		Multivariable	
	HR (95% CI)	p Value	HR (95% CI)	p Value
Age, year	1.09 (1.03–1.14)	<0.01	1.05 (1.00–1.11)	0.09
BMI, kg/m^2^	0.96 (0.85–1.09)	0.57		
PSA level, ng/mL	0.99 (0.97–1.01)	0.28		
Biopsy Gleason score		0.03		0.03
≤8	1(ref)		1(ref)	
≥9	1.94 (1.08–3.53)		1.91 (1.05–3.47)	
Number of positive biopsy core	0.96 (0.87–1.06)	0.44		
Percentage of positive biopsy core, %	0.99 (0.98–1.00)	0.15		
Clinical T stage		0.65		
≤T2	1(ref)			
≥T3	0.87 (0.47–1.61)			
ACCI		<0.01		<0.01
≤3	1(ref)		1 (ref)	
>3	3.52 (1.91–6.51)		2.71 (1.36–5.40)	

BMI = body mass index; ACCI = age adjusted Charlson Comorbidity Index; PSA = prostate specific antigen

Univariable and multivariable Cox regression analyses were performed for OS. OS was significantly correlated with biopsy Gleason Score (HR 1.91, 95% CI 1.05–3.47) and ACCI score >3 (HR 2.71, 95% CI 1.36–5.40). PSA level and clinical T stage did not correlate with OS.

**Fig 1:**
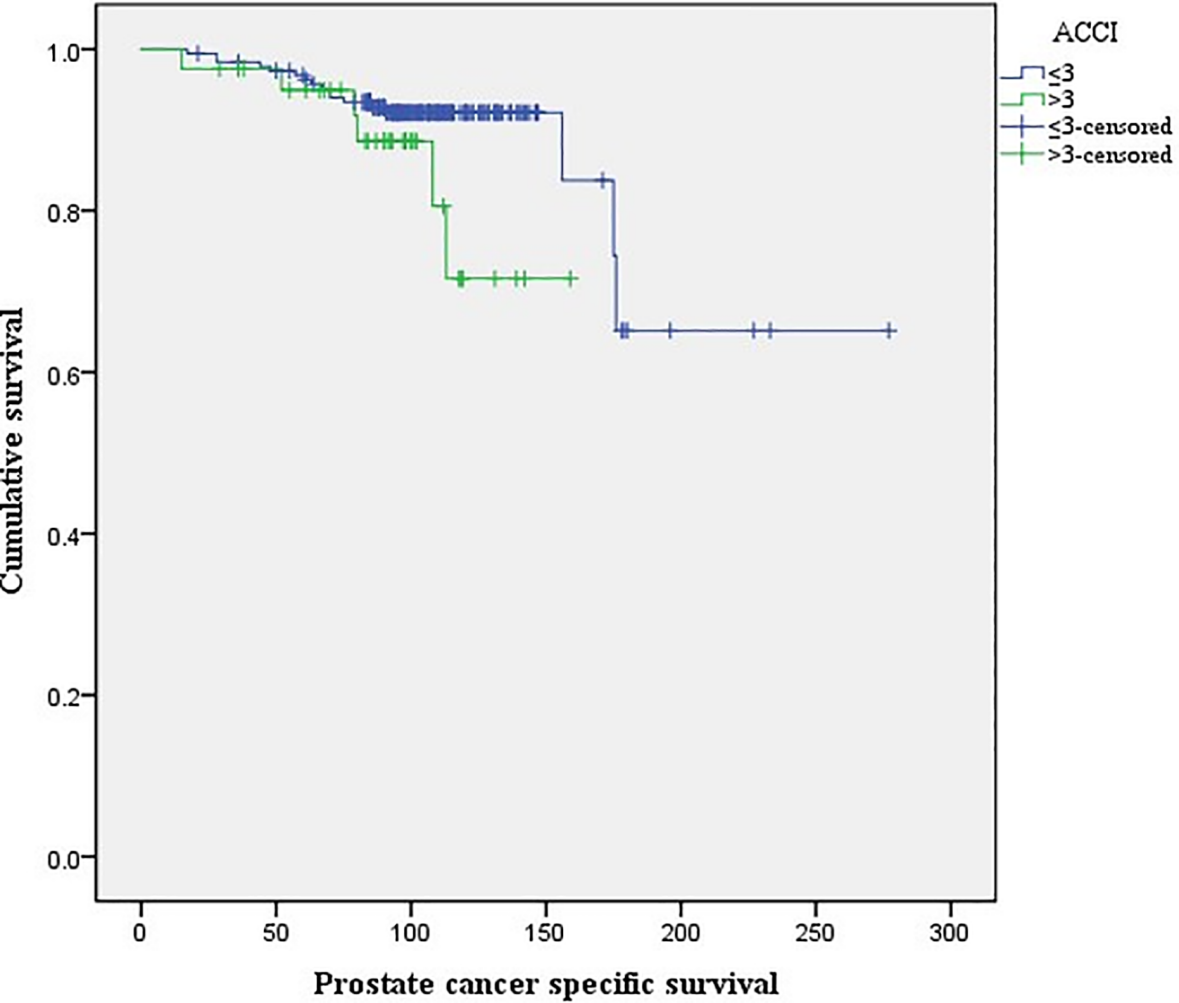
Kaplan-Meier curves of prostate cancer-specific for high ACCI (>3) and low ACCI (≤3) groups (log rank test; p = 0.09).

**Fig 2:**
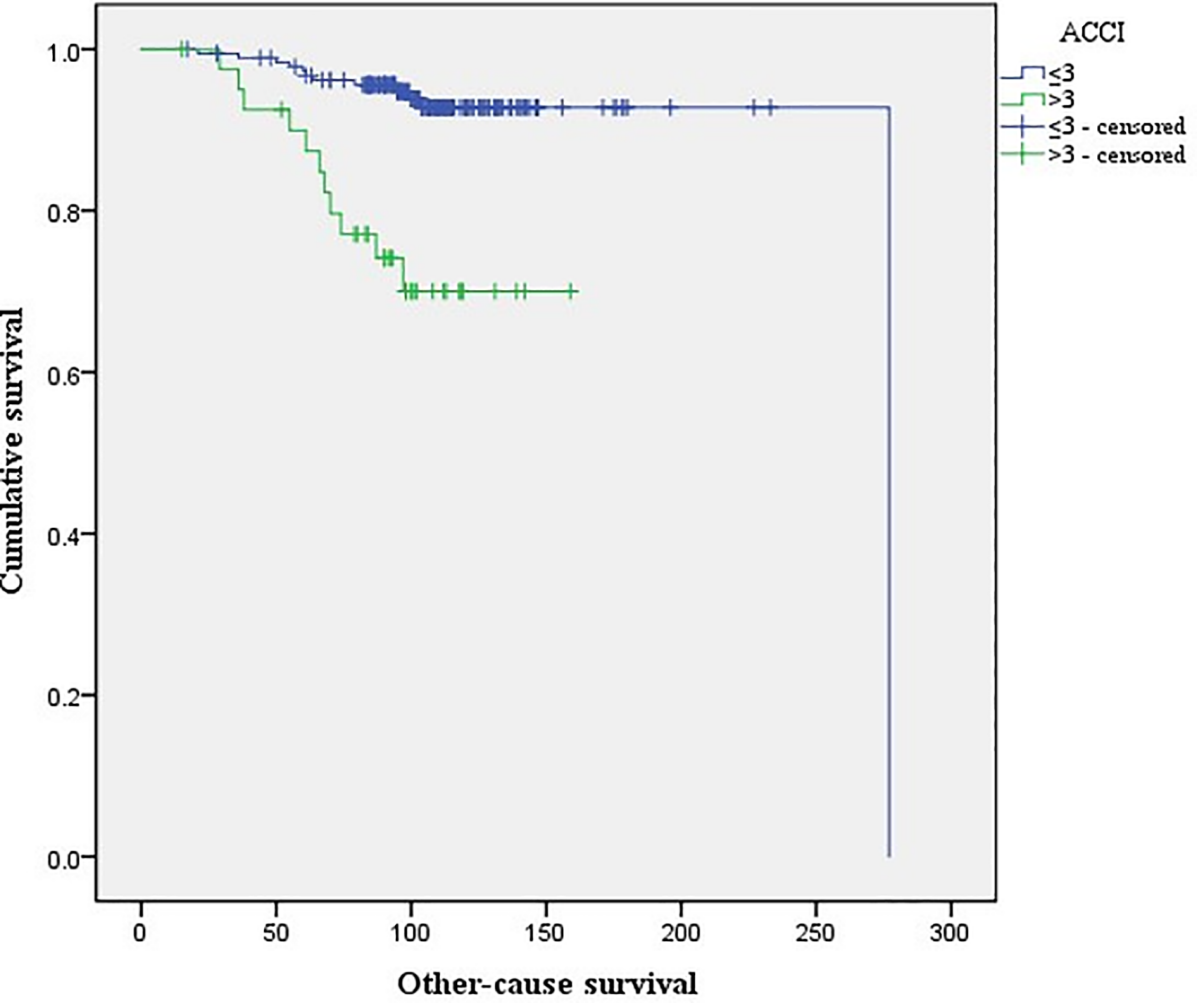
Kaplan-Meier curves of other-cause survival for high ACCI (>3) and low ACCI (≤3) groups (log rank test; p<0.01).

**Fig 3:**
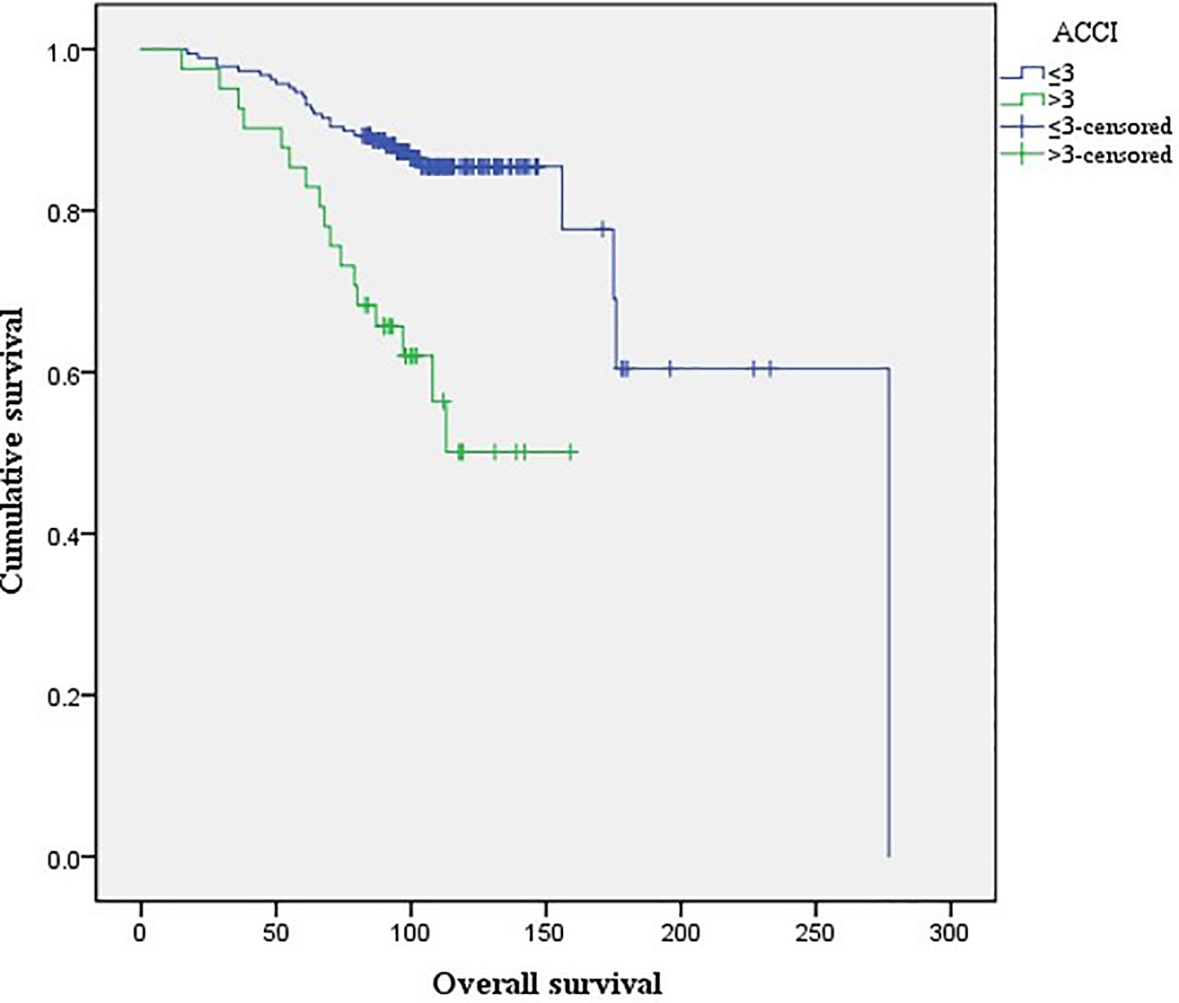
Kaplan-Meier curves of overall survival for high ACCI (>3) and low ACCI (≤3) groups (log rank test; p< 0.01).

There were no differences in the Kaplan-Meier curves of PCSS between the high (ACCI >3) and low ACCI groups (ACCI ≤3; log rank test, p = 0.09; Fig 1). Kaplan-Meier curves for OCS and OS revealed that for both measures, the high ACCI group had lower OCS (p<0.01; Fig 2) and OS (p<0.01; Fig 3) rates than the low ACCI group. Together, these data suggest that ACCI can be used as a predictor of patient survival after RP.

## Discussion

In this study, we identified a new prognostic factor for predicting survival after RP in patients with PC. PC is a devastating condition that is currently the most prominent type of newly diagnosed cancers and the third highest cause of cancer-related deaths in males [1]. Although early stage PC is relatively benign and may not progress under active surveillance [17], advanced PC can become refractory to all known treatments and can be lethal [3]. Thus, research involving PC risk and outcomes is highly relevant. However, PC is a heterogeneous disorder that can be difficult to classify. To contend with this heterogeneity, researchers have developed a risk classification system to identify the risk of PC. Previously, D’Amico et al. suggested that risk classification should be done according to PSA levels, clinical T stage, and biopsy Gleason score [2]. However, patient clinical outcomes varied greatly within a high-risk PC group when classified by biopsy Gleason score or clinical T stage [18]. Researchers have since suggested that high-risk PC should be further stratified into high- or very high-risk PC [3]. Compared with the standard high-risk PC group, patients with very high-risk PC had significantly worse BCR-free survival, distant metastasis-free survival, and PC-specific mortality [3].

There are many treatment options for PC depending on patient risk classification. For very high-risk PC patients, NCCN guidelines recommend external beam radiotherapy or RP as potential therapies [4]. Fortunately, radical local treatments including surgery or radiotherapy decrease mortality in men with very high-risk PC. Stattin et al. reported that very high-risk PC patients who receive RP or full-dose radiotherapy had lower PC-associated and overall mortality [19]. Thus, RP is a feasible treatment option for very high-risk PC and could result in long-term progression-free survival [9, 19]. Very high-risk PC patients treated with RP also had favorable metastatic progression-free survival, PC-specific mortality-free survival, and OS rates despite receiving a relatively poor prognosis. Surprisingly, their functional outcomes were not any worse than those of standard high-risk PC patients according to Pompe et al [20]. This suggests that RP is an effective treatment option for men with very high-risk PC.

In this study, we sought to identify whether ACCI could serve as a new prognostic factor for the efficacy of RP as a treatment for very high-risk PC, as clinicians consider patient comorbidities when selecting treatments for patients with PC [21] and typically treat patients with a comorbidity less aggressively than those without a comorbidity [22]. A more accurate risk classification system could improve treatment for older patients without significant comorbidities [23]. Patient comorbidity is also an important independent prognostic factor for cancer outcomes [10]. Indeed, Post et al. reported that comorbidity was the most important prognostic factor for 3-year survival among 1,337 localized-PC patients under the age of 75 years [24]. Although use of the optimal comorbidity index is controversial [25], the CCI is still widely used when selecting treatment options for PC [21, 26, 27].

In a previous study, Lund et al.reported comorbidities in more than one-third of 8,114 newly diagnosed PC patients, with patients with higher CCI scores having higher 1-year mortality rates [28]. Separately, Kastner et al. reported that the CCI was a significant predictor of survival following radical treatment in 37 localized PC patients [26]. After RP, age-adjusted CCI was a significant prognostic factor for long-term survival of patients with high-risk PC. Lee et al. investigated 542 PC patients who underwent RP and reported that ACCI was the most powerful predictor of overall and non-PC-related mortality [29]. In another study, Lee et al. showed that the CCI was associated with OS and non-PCSS in 335 Korean men who underwent RP for PC [30]. However, this study did not identify the optimal selection criteria for surgical treatment of patients with very high-risk PC. Current NCCN guidelines only recommend RP for very high-risk PC patients who are young and healthy [4]. However, optimal criteria for the selection of these young and healthy patients are limited. Thus, we attempted to suggest criteria for classification of younger and healthier patients when considering RP. In the current study, we assessed the accuracy of the ACCI as a selection criteria for RP in older patients with very high-risk PC. We compared different survival measures between high CCI (>3) and low CCI (≤3) groups. As expected, patients with a low ACCI showed significantly more favorable OCS and OS compared with those with a high ACCI. These data support the use of RP for suitable older patients with very high-risk PC.

This study has several limitations. First, analysis was performed retrospectively with a relatively small sample size within a single institution. Thus, a multicenter, prospective study with a larger sample size is needed to generalize this hypothesis. Second, this study included only patients who received RP. Often, healthier patients are selected to undergo surgical treatment rather than a non-surgical treatment such as radiotherapy, which may introduce selection bias. Finally, we only compared patient outcomes between high and low ACCI groups after RP. Future studies should compare very high-risk PC patient outcomes following different treatments or between surgical and non-surgical treatment groups.

Despite these limitations, we show that the ACCI could be a prognostic factor for very high-risk PC patients and should be used when considering surgical treatments for PC. Older or ill patients with an ACCI score >3 showed significantly worse survival outcomes after RP. This is the first study to identify a threshold ACCI for use when considering RP for patients with very high-risk PC, which can aid clinicians making treatment decisions for such patients.

## Conclusion

Patient comorbidities should be considered when selecting treatments for PC. The ACCI is a feasible prognostic factor for non-PCSS and OS in very high-risk PC patients. RP should be considered carefully for patients with an ACCI score >3.
